# Supplementary material for: Simultaneous atmospheric water production and 24-hour power generation enabled by moisture-induced energy harvesting
Source: Nat Commun. 2022 Nov 9;13:6771. doi: 10.1038/s41467-022-34385-4 (PMC9646691; doi:10.1038/s41467-022-34385-4)
Supplement: Supplementary file 1 — Supplementary Information [file 41467_2022_34385_MOESM1_ESM.pdf]

Supplementary Information

For

Simultaneous Atmospheric Water Production and 24-hour Power Generation Enabled by Moisture-induced Energy Harvesting

Tingxian Li[1,2,†,*], Minqiang Wu[1,†], Jiaxing Xu[1,†], Ruxue Du[1], Taisen Yan[1], Pengfei Wang, Zhaoyuan Bai[1], Ruzhu Wang[1,2] and Siqi Wang[1]

[1]Institute of Refrigeration and Cryogenics, School of Mechanical Engineering, Shanghai Jiao Tong University, Shanghai, 200240, China

[2]Research Center of Solar Power and Refrigeration of Ministry of Education, Shanghai Jiao Tong University, Shanghai, 200240, China

[†]These authors contribute equally to this work

*Corresponding author. E-mail: Litx@sjtu.edu.cn

Table of Contents

Supplementary Notes

Supplementary Note 1: Theoretical analysis of the energy balance of hybrid SAWH-TEPG for water and power co-generation

Supplementary Note 2: Calculation of the radiative cooling power, sorption heating power, desorption cooling power and convective heat loss

Supplementary Note 3: Preparation of MIL-101(Cr)@CF composite

Supplementary Note 4: Characterizations of MIL-101(Cr)@CF composite

Supplementary Note 5: Water sorption of MIL-101(Cr)@CF composite

Supplementary Note 6: Design and fabrication of hybrid SAWH-TEPG device

Supplementary Note 7: Performance of hybrid SAWH-TEPG device. (i) Indoor experiments, (ii) Outdoor experiments

List of Figures and Tables

Supplementary Figure 1. (a) Energy balance model and (b) theoretical thermal equilibrium analysis of the hybrid SAWH-TEPG device during daytime. (c) Energy balance model and (d) theoretical thermal equilibrium analysis of the hybrid SAWH-TEPG device during nighttime.

Supplementary Figure 2. (a) Water sorption of various sorbents at typical climates at 70% RH at 30 °C (~3.0 kPa) (b) The isothermal equilibrium curves of reported sorbents.

Supplementary Figure 3. Variable-temperature water vapor sorption isotherms of pristine MIL-101(Cr) powder at 15°C, 25°C, and 35°C.

Supplementary Figure 4. N_2 adsorption isotherms of synthetic MIL-101(Cr) powder at 77K.

Supplementary Figure 5. Sorption kinetics of MIL-101(Cr)@CF composite at different RH.

Supplementary Figure 6. Water uptake of MIL-101(Cr) powder and MIL-101(Cr)@CF composite samples at typical 25 °C and 65% RH.

Supplementary Figure 7. IR images of MIL-101(Cr)@CF samples during moisture sorption process at different times.

Supplementary Figure 8. Temperature evolution, water uptake of MIL-101(Cr)@CF samples and ambient condition during moisture sorption process.

Supplementary Figure 9. Mass changes of MIL-101(Cr)@CF during shaky condition simulated by an 80 W ultrasonic cleaner.

Supplementary Figure 10. Powder X-ray diffraction (PXRD) of pristine MIL-101(Cr), MIL-101(Cr) with binder, and MIL-101(Cr)@CF after 20 sorption-desorption cycles.

Supplementary Figure 11. FTIR patterns of pristine MIL-101(Cr) powder, MIL-101(Cr) with binder, and MIL-101(Cr)@CF after 20 sorption-desorption cycles.

Supplementary Figure 12. Experimental setup of home-made test rig in the environment-controlled chamber.

Supplementary Figure 13. Output voltage, current and power of the TEPG module, under the condition of 2 °C temperature difference.

Supplementary Figure 14. Variation of ambient condition within a typical experimental period in the test chamber.

Supplementary Figure 15. Comparison of maximum output voltages of three different experimental devices.

Supplementary Figure 16. Photograph of hybrid SAWH-TEPG device for indoor experiments under simulated sunlight.

Supplementary Figure 17. Maximum output power densities of the hybrid SAWH-TEPG device under different simulated sunlight intensities.

Supplementary Figure 18. Comparison of radiative cooling power of the hybrid SAWH-TEPG devices with MOF and the referenced device without MOF during nighttime.

Supplementary Figure 19. Comparison of sorption heating power, radiative cooling

power, and convective heat loss of the hybrid SAWH-TEPG devices at 22:30 pm and 1:30 am during nighttime.

Supplementary Figure 20. Comparison of maximum output power of the hybrid SAWH-TEPG devices with MOF and the referenced device without MOF during nighttime.

Supplementary Figure 21. (a) Water uptake, ambient temperature, and ambient RH changes of the device during the sorption process. (b) Sorption heating power of the sorbent.

Supplementary Figure 22. (a) Theoretical thermal equilibrium results of the hybrid SAWH-TEPG device at (a) 12:30 pm and (b) 14:00 pm during daytime.

Supplementary Figure 23. Maximum output power of hybrid SAWH-TEPG device with MOF during daytime.

Supplementary Figure 24. (a) Mass, ambient temperature, and ambient RH changes of the device during the desorption process. (b) Desorption cooling power of the sorbent.

Supplementary Figure 25. Photograph of air-cooled condenser using aluminum flat fins.

Supplementary Figure 26. (a) Variation of annual average solar irradiation intensity in one day. (b) Variation of annual average relative humidity in one day.

Supplementary Figure 27. Predicted annual water production of the SAWH-TEPG device in different cities.

Supplementary Figure 28. Solar irradiation, air RH, and ambient temperature during a continuous week of outdoor experiments.

Supplementary Table 1. Comparison of power density of different simultaneous water and electricity generation systems.

Supplementary Note 1.

Theoretical analysis of the energy balance of hybrid SAWH-TEPG for water and electricity co-generation

The structure of the SAWH-TEPG device is schematically shown in Figure 1a, 1b. During daytime, the device is closed and the SAWH-TEPG device is exposed to solar irradiance for water releasing (desorption). The solar energy is first converted into thermal heat and then transferred from the solar absorber to the sorbent through the TEPG module. The desorbed water vapor diffuses from the sorbent layer to the condenser due to a concentration gradient. Accumulation of vapor in the enclosure leads to saturation conditions and consequently, the condensation process occurs at ambient temperature. The heat of condensation is dissipated to the ambient by a heat sink. The energy balance model of this device during solar-driving water desorption and harvesting process can be expressed as:

$$Q_{\mathrm{sol}} = Q^{\mathrm{i}}_{\mathrm{rad,abs}} + Q^{\mathrm{i}}_{\mathrm{conv,abs}} + Q^{\mathrm{i}}_{\mathrm{ele}} + Q^{\mathrm{i}}_{\mathrm{rad,sor}} + Q^{\mathrm{i}}_{\mathrm{conv,sor}} + Q^{\mathrm{i}}_{\mathrm{des}} \quad (1)$$

where Q_{sol} is the absorbed solar energy, $Q^{\mathrm{i}}_{\mathrm{ele}}$ is the converted electrical power, $Q^{\mathrm{i}}_{\mathrm{rad,abs}}$ and $Q^{\mathrm{i}}_{\mathrm{conv,abs}}$ are the radiative and convective heat loss of the dual-functional coating layer, $Q^{\mathrm{i}}_{\mathrm{rad,sor}}$ and $Q^{\mathrm{i}}_{\mathrm{conv,sor}}$ are the radiative and convective heat loss of the sorbent, $Q^{\mathrm{i}}_{\mathrm{des}}$ desorption heat of the sorbent, which can be calculated as the product of the desorption rate ($\dot{m}$, g s^{-1}) and the desorption enthalpy (h_{des}, J g_{water}^{-1}): $Q^{\mathrm{i}}_{\mathrm{des}} = \dot{m}_{\mathrm{des}} h_{\mathrm{des}}$. As a result, the thermal heat passing through the hot-side and cold-side of TEPG module ($Q^{\mathrm{i}}_{\mathrm{TEG}}$) are determined by:

$$Q^{\mathrm{i}}_{\mathrm{TE}} = Q^{\mathrm{i}}_{\mathrm{rad,sor}} + Q^{\mathrm{i}}_{\mathrm{conv,sor}} + Q^{\mathrm{i}}_{\mathrm{des}} \quad (2)$$

$$Q^{\mathrm{i}}_{\mathrm{TE}} = A_1 \sigma \varepsilon_{\mathrm{sor}} \left({T^{\mathrm{i}}_{\mathrm{sor}}}^4 - {T^{\mathrm{i}}_{\mathrm{cond}}}^4 \right) - Ah\left(T^{\mathrm{i}}_{\mathrm{sor}} - T^{\mathrm{i}}_{\mathrm{cond}}\right) + \dot{m}_{\mathrm{des}} h_{\mathrm{des}} \quad (3)$$

where A_1 is the area of sorbent, σ is the Stefan-Boltzmann constant, $\varepsilon_{\mathrm{sor}}$ is the

emissivity of the sorbent, $T_{\mathrm{sor}}^{\mathrm{i}}$ and $T_{\mathrm{amb}}^{\mathrm{i}}$ are the temperatures of the sorbent and the ambient, h is the conductive heat transfer coefficient. The thermal resistance networks. According to heat transfer characteristics, the $\mathrm{Q}_{\mathrm{TE}}^{\mathrm{i}}$ can also be calculated as:

$$Q_{\mathrm{TE}}^{\mathrm{i}} = A_2 \frac{\left(T_{\mathrm{hot}}^{\mathrm{i}} - T_{\mathrm{cold}}^{\mathrm{i}}\right)}{R_{\mathrm{TE}}} = A_2 \frac{\Delta T_{\mathrm{TE}}}{R_{\mathrm{TE}}} \tag{4}$$

where A_2 is the area of TEPG module, R_{TE} is the thermal resistance of the TEPG-module, $T_{\mathrm{hot}}^{\mathrm{i}}$ and $T_{\mathrm{cold}}^{\mathrm{i}}$ are the hot-side and cold-side temperatures of TEPG module. Additionally, the maximum electrical power of a TEPG module (P_{max}) is given by the following equation:

$$P_{\mathrm{max}} = \frac{\left(n(S_{\mathrm{pn}})\Delta T_{\mathrm{TE}}\right)^2}{4R_{\mathrm{L}}} \tag{5}$$

where n is the number of thermoelements, R_{L} is the loading resistance, S_{pn} is the Seebeck coefficient of TEPG materials. Notably, in addition to the intrinsic merit of the TEPG material and the R_{L}, the theoretical maximum output power (P_{max}) depends only on the temperature difference of the TEPG module (ΔT_{TE}).

Compared with the conventional solar-driven thermoelectric device where the residual thermal heat is mainly released through natural convection and radiative heat loss, the introduction of sorbent leads to a huge thermal consumption ($Q_{\mathrm{des}}^{\mathrm{i}}$) owing to the water desorption process. Thus, the cold-side temperature of TEPG module decreases from $T_{\mathrm{cold,non}}^{\mathrm{i}}$ to $T_{\mathrm{cold}}^{\mathrm{i}}$ and the resultant temperature difference of TEPG module enlarges ($\Delta T_{\mathrm{TE,non}}^{\mathrm{i}}$ to $\Delta T_{\mathrm{TE}}^{\mathrm{i}}$). As a result, the combination of sorbent leads to an increment in the maximum output power of TEPG module.

During nighttime, the proposed SAWH-TEPG device is opened and the sorbent is exposed to the ambient to saturate with vapor from the natural flow of air and passively cooled with radiation to the sky. Radiative cooling is a passive cooling technique that

cools object by radiating a fraction of the object's thermal radiation to the cold of outer space. Different from the conventional AWH device, in which a considerable amount of sorption heat is wasted through nature convection, the radiative cooling surface of our device is applied as the cold-side of the TEPG module, while the hot-side is attached with a sorbent and heated through the sorption heat. Thus, a temperature difference is passively created and electricity can be generated by the TEPG. The energy balance model during radiative cooling and water sorption process can be described as:

$$Q_{\mathrm{ads}}^{\mathrm{ii}} = Q_{\mathrm{conv,sor}}^{\mathrm{ii}} + Q_{\mathrm{rad,sor}}^{\mathrm{ii}} + Q_{\mathrm{ele}}^{\mathrm{ii}} + Q_{\mathrm{conv,abs}}^{\mathrm{ii}} + Q_{\mathrm{rad}} \tag{6}$$

where $Q_{\mathrm{ads}}^{\mathrm{ii}}$ is the sorption heat, which can be calculated as the product of the adsorption rate ($\dot{m}_{\mathrm{ads}}$, g s^{-1}) and the adsorption enthalpy (h_{ads}, J g_{water}^{-1}): $Q_{\mathrm{ads}}^{\mathrm{ii}} = \dot{m}_{\mathrm{ads}} h_{\mathrm{des}}$. $Q_{\mathrm{conv,sor}}^{\mathrm{ii}}$ and $Q_{\mathrm{rad,sor}}^{\mathrm{ii}}$ are the radiative and convective heat loss of the sorbent, $Q_{\mathrm{ele}}^{\mathrm{ii}}$ is the converted electrical power, Q_{rad} and $Q_{\mathrm{conv,abs}}^{\mathrm{ii}}$ are the radiative and convective heat loss of the dual-functional coating layer. Similarly, the thermal heat passing through the hot-side and cold-side of TEPG module ($Q_{\mathrm{TE}}^{\mathrm{ii}}$) are determined by:

$$Q_{\mathrm{TE}}^{\mathrm{ii}} = Q_{\mathrm{conv,abs}}^{\mathrm{ii}} + Q_{\mathrm{rad}} \tag{7}$$

$$Q_{\mathrm{TE}}^{\mathrm{ii}} = A_3 \sigma \varepsilon_{\mathrm{abs}} \left({T_{\mathrm{abs}}^{\mathrm{ii}}}^4 - {T_{\mathrm{amb}}^{\mathrm{ii}}}^4 \right) - Ah\left(T_{\mathrm{abs}}^{\mathrm{ii}} - T_{\mathrm{amb}}^{\mathrm{ii}}\right) \tag{8}$$

Where A_3 the area of dual-functional coating layer, $\varepsilon_{\mathrm{abs}}$ is the emissivity of the dual-functional coating layer, $T_{\mathrm{abs}}^{\mathrm{ii}}$ and $T_{\mathrm{amb}}^{\mathrm{ii}}$ are the temperatures of the dual-functional coating layer and the ambient, h is the conductive heat transfer coefficient. According to heat transfer characteristics, the $Q_{\mathrm{TE}}^{\mathrm{ii}}$ can also be calculated as:

$$Q_{\mathrm{TE}}^{\mathrm{ii}} = A_2 \frac{\left(T_{\mathrm{hot}}^{\mathrm{ii}} - T_{\mathrm{cold}}^{\mathrm{ii}}\right)}{R_{\mathrm{TE}}} = A \frac{\Delta T_{\mathrm{TE}}}{R_{\mathrm{TE}}} \tag{9}$$

where $T_{\mathrm{hot}}^{\mathrm{ii}}$ and $T_{\mathrm{cold}}^{\mathrm{ii}}$ are the hot-side and cold-side temperatures of TEPG module.

The electric power is generated through the combination of adsorption heating and

radiative cooling. Compared with conventional radiative cooling-based thermoelectric electricity generation, the synergistic effect of sorption heating and passive cooling is able to enlarge the hot-side temperature of TEPG module ($T_{\mathrm{hot,non}}^{\mathrm{ii}}$ to $T_{\mathrm{hot}}^{\mathrm{ii}}$) and promote the temperature difference between hot-side and cold-side of TEPG module ($\Delta T_{\mathrm{TE,non}}^{\mathrm{ii}}$ to $\Delta T_{\mathrm{TE}}^{\mathrm{ii}}$), thus improves the electricity generation performance. Additionally, compared with the naturally cooled AWH devices, the passive radiative cooling facilitates the dissipation of sorption heat, which lowers the sorbent temperature and increases water uptake, leading to a significant improvement in water harvesting output of the SAWH device.

Supplementary Note 2.

Calculation of radiative cooling power, sorption heating power and desorption cooling power

A thermal model is developed to analyze the performance of the SAWH-TEPG device (Supplementary Figure 1). The energy balance model of the SAWH-TEPG device during daytime and nighttime can be expressed as:

Daytime:

$$P_{\mathrm{des}} = P_{\mathrm{solar}} - P_{\mathrm{rad}}(T_{\mathrm{abs}}) + P_{\mathrm{atm}}(T_{\mathrm{amb}}) - P_{\mathrm{conv}}^{\mathrm{abs}}(T_{\mathrm{abs}}, T_{\mathrm{amb}}) - P_{\mathrm{conv}}^{\mathrm{sor}}(T_{\mathrm{abs}}, T_{\mathrm{amb}}) = \frac{\dot{m}_{\mathrm{MOF}} \cdot \Delta H_{\mathrm{H_2O}}}{M_{\mathrm{H_2O}}} \tag{10}$$

Nighttime:

$$P_{\mathrm{sor}} = P_{\mathrm{rad}}(T_{\mathrm{abs}}) - P_{\mathrm{atm}}(T_{\mathrm{amb}}) + P_{\mathrm{conv}}^{\mathrm{abs}}(T_{\mathrm{abs}}, T_{\mathrm{amb}}) + P_{\mathrm{conv}}^{\mathrm{sor}}(T_{\mathrm{abs}}, T_{\mathrm{amb}}) = \frac{\dot{m}_{\mathrm{MOF}} \cdot \Delta H_{\mathrm{H_2O}}}{M_{\mathrm{H_2O}}} \tag{11}$$

$$P_{\mathrm{cool}}^{\mathrm{net}} = P_{\mathrm{rad}} - P_{\mathrm{atm}}(T_{\mathrm{amb}}) \tag{12}$$

where P_{des} is the desorption cooling power, P_{solar} is the incident solar irradiation

absorbed by the absorber, P_{sor} is the sorption heating power, $P_{\text{rad}}(T_{\text{abs}})$ is the power radiated by the absorber, $P_{\text{atm}}(T_{\text{amb}})$ is the absorbed atmospheric thermal radiation, $P_{\text{conv}}^{\text{abs}}(T_{\text{abs}}, T_{\text{amb}})$ is the convective power lost by the absorber, $P_{\text{conv}}^{\text{sor}}(T_{\text{abs}}, T_{\text{amb}})$ is the convective power lost by the sorbent, $P_{\text{cool}}^{\text{net}}$ is the net radiative cooling power of the absorber. $\dot{m}_{\text{MOF}}$ is the mass change rate (water sorption or desorption rate) of the MOF sorbent, $\Delta H_{\text{H}_2\text{O}}$ is the water sorption enthalpy (44 kJ mol^{-1} water), $M_{\text{H}_2\text{O}}$ is the relative molecular mass of water.

In the energy balance models, we approximate that the total heat flows are dominated by sorption heat and neglect heat generation and absorption due to the Seebeck effect and Joule heating in the thermoelectric legs. This simplifying approximation is made due to the power conversion efficiency of the thermoelectric module is expected to be well below 0.5%. Therefore, the above parameters can be calculated by the following equations:

$$P_{\text{solar}} = \int d\lambda \varepsilon(\lambda, \theta_{\text{solar}}) I_{\text{AIM1.5}}(\lambda) \tag{13}$$

$$P_{\text{rad}}(T_{\text{abs}}) = \int d\Omega cos\theta \int_0^{\infty} d\lambda I_{\text{BB}}(T_{\text{abs}}, \lambda)\, \varepsilon(\lambda, \theta) \tag{14}$$

$$P_{\text{atm}}(T_{\text{amb}}) = \int d\Omega cos\theta \int_0^{\infty} d\lambda I_{\text{BB}}(T_{\text{amb}}, \lambda)\, \varepsilon(\lambda, \theta) \varepsilon_{\text{atm}}(\lambda, \theta) \tag{15}$$

$$P_{\text{conv}}^{\text{abs}}(T_{\text{abs}}, T_{\text{amb}}) = h_{\text{abs}}(T_{\text{abs}} - T_{\text{amb}}) \tag{16}$$

$$P_{\text{conv}}^{\text{sor}}(T_{\text{abs}}, T_{\text{amb}}) = h_{\text{sor}}(T_{\text{sor}} - T_{\text{amb}}) \tag{17}$$

where $\int d\Omega = 2\pi \int_0^{2\pi} d\theta sin\theta$ is the angular integral over a hemisphere. $I_{\text{BB}}(T, \lambda) = \frac{2hc^2}{\lambda^5} \frac{1}{e^{hc/(\lambda k_{\text{B}} T)-1}}$ is the spectral radiance of a blackbody at temperature *T*, *h* is Planck's constant, κ_B is the Boltzmann constant, and *c* is the light speed. $\varepsilon(\lambda, \theta)$ is the directional emissivity of the surface at wavelength λ. For simplicity, solar absorber (emitter) can be

approximated as a graybody with average emissivity $\varepsilon_s = 0.95$ we can express the above equations as $P_{rad}(T_{abs}) = \varepsilon_s \sigma T_{abs}^4$ and $P_{atm}(T_{amb}) = \varepsilon_{atm} \varepsilon_s \sigma T_{abs}^4$. The ε_{atm} can be defined by the correlation of $\varepsilon_{atm} = 0.741 + 0.0062 T_{dew}$. T_{dew} is the dew point temperature. h_{abs} and h_{sor} are the convective heat transfer coefficients of the absorber and the sorbent, and they are assumed to be 5 W m^{-2} K^{-1} according to the literatures [1-3].

Supplementary Note 3.

Preparation of MIL-101(Cr)@CF composite

Chemicals including chromium nitrate ($Cr(NO_3)_3$ $9H_2O$), 1,4-benzenedicarboxylic acid (H_2BDC), N,N-Dimethyl formamide (DMF), and ethanol were purchased from Sigma-Aldrich. MIL-101(Cr) nanoparticles were synthesized based on a reported procedure [4]. At first, 1 mmol chromic chloride hexahydrate and 1 mmol terephthalic acid were dissolved into 7.2 mL deionized water. Subsequently, the mixed solution was vigorously stirred for complete dissolution before hydrothermal treatment at 190 ºC for 24 h. After the reaction finished, the resultant solution was naturally cooling down to room temperature, the recrystallized terephthalic acid was removed by low-speed centrifugation (350 g, 3 min). Next, the MIL-101(Cr) product in the green color supernatant was collected by centrifugation (7700 g, 15 min), washing with DMF and ethanol respectively, and drying at 80 °C for 4 h.

Copper foams (50 × 50 × 5 mm and 100 × 100 × 5 mm) were immersed in 0.5 mol L^{-1} HCl solution until the surface color turned red, and then immersed in ethanol and deionized water alternatively for ultra-sonic cleaning 5 min. Afterwards, the clean copper foam was dried at 50 °C. 1 g prepared MIL-101(Cr) powders were mixed with

8mL ethanol and 2 mL deionized water. The suspension was homogenized in an ultra-sonic bath for 20 min, and 0.37 g silicate sol (~30 wt%) was added as binder [5]. The prepared copper foam was immersed in the as-synthesized MOF suspension slurry and then dried at 80 °C for 24 h. A robust MOF coating forms on the skeleton of porous CF with a high mass fraction of MIL-101(Cr) up to 90wt %.

Supplementary Note 4.

Characterizations of MIL-101(Cr)@CF composite

The morphology of MIL-101(Cr) and the MIL-101(Cr)@CF composite were observed by Scanning Electron Microscopy (SEM, Sirion 200 instrument, FEI) equipped with an energy-dispersive X-ray spectrometer (EDS, INCA X-Act attachment, Oxford). The nitrogen adsorption of synthesized MIL-101(Cr) was measured by the volumetric method using the physisorption apparatus (Autosorb-IQ3, Quantachrome) at 77K followed by the evacuated degas at 393K for 12h. The specific surface area is determined to be 2850 g cm^{-3} by the Brunauer-Emmett-Teller (BET) method. The thermal conductivities of the MIL-101(Cr) powder, MIL-101(Cr) tablet and MIL-101(Cr)@CF composite were measured by the laser flash method (LFA 447, Netzsch) and Hot Disk thermal constants analyzer (TPS3500, Hot Disk AB Company, Sweden). The PXRD patterns were measured by an X-ray diffractometer (Ultima IV, Rigaku). FT-IR spectra were measured by an FT-IR spectrometer (Nicolet 6700, Thermo Fisher Scientific) The water sorption isotherms were measured using an accelerated surface area and porosimetry analyzer (ASAP2020, Micromeritics) under a water vapor atmosphere with controllable vapor pressure. The sample temperature was set as a constant (15, 25, and 35 °C), and the relative pressure of the water vapor was increased from 0 to 1 according to a set of pressure intervals. The TGA tests were carried out by a commercial

thermogravimetric analyzer (STA 449, Netzsch) equipped with a moisture humidity generator (MHG 32, ProUmid).

Supplementary Note 5.

Water sorption of MIL-101(Cr)@CF composite

Water adsorption capacities of the MOF sorbent and its composite were studied by a gravimetric method using a precision electronic balance (ME503TE, METLER TOLEDO) with a resolution of 1 mg. Prior to experiments, the MOF sorbent and its composite samples were dried at 100 °C for 4h to determine the dry MOF coating mass, $m_{coating}$ (g). The mass fraction of the pure MOF sorbent in the coating can be calculated based on the slurry recipe. The coated sample was then cooled down and placed on the balance at a predetermined environment condition. The mass change Δm (g) during the adsorption process was recorded continuously until equilibrium. The water adsorption capacity of the MOF sorbent (in $g_{water}\ g_{coating}^{-1}$) is calculated as:

$$W_{\mathrm{MOF}} = \frac{\Delta m}{m_{\mathrm{coating}}} \tag{18}$$

Meanwhile, the water adsorption of the MIL-101(Cr) based on the mass of pure sorbent (in $g_{water}\ g_{sorbent}^{-1}$) is calculated as:

$$W_{\mathrm{MOF}} = \frac{\Delta m}{m_{\mathrm{coating}} - m_{\mathrm{binder}}} = \frac{\Delta m}{m_{\mathrm{MOF}}} \tag{19}$$

where m_{binder}, m_{MOF} are the mass of the binder and the pure sorbent in the MOF coating (g), respectively. This parameter can be used to indicate the possible influence of the binder on the original adsorption capacity of the sorbent.

Supplementary Note 6.

Design and fabrication of hybrid SAWH-TEPG device

For proof-of-concept experiments, we fabricate a hybrid SAWH-TEPG device by

assembling MOF sorbent with a TEPG module. Specifically, the hybrid SAWH-TEPG device consists of four parts: a square wall made from transparent acrylic tube (length × width × thinness 20 cm × 20 cm × 1 cm), a copper foam (CF) block adhered with MIL-101(Cr) powder (length × width × thinness 100 × 100 × 5 mm, MOF sorbent weight: 8.1 g), a commercial TEPG module (length × width × thinness 40 × 40 × 4 mm) and a copper plate painted with a commercial black paint (length × width × thinness 100 × 100 × 0.8 mm). The top of the hybrid SAWH-TEPG device is enclosed with an infrared-transparent wind cover made from 12.5 mm-thick low-density polyethylene previously to reduce the heat loss during both daytime and night. In the hybrid SAWH-TEPG device, the TEPG module is embedded in SAWH module, placed between a dual-functional coating layer and MIL-101(Cr)@CF through a small aluminum block as thermal transport framework. The dual-functional coating layer works as light-to-heat absorber for radiative heating in daytime or thermal emitter for radiative cooling in nighttime. Moreover, the working modes of dual-functional coating layer can be automatically switched between absorber and emitter owing to its blackbody radiation characteristics. The employed thermoelectric generator not only converts the excessive solar-thermal energy to be electricity during daytime but also enables electricity generation by simultaneously harvesting coldness from the sky and obtaining sorption heat during water vapor capture of MOF at night.

Supplementary Note 7.

Performance of hybrid SAWH-TEPG device

(i) Indoor experiments

For nighttime proof-of-concept experiments, in prior to the experiments, the hybrid SAWH-TEPG device undergoes a thorough desorption stage at 85 °C for 5h. Then, we

placed the device in an environment-controlled chamber with 65% RH and 25 °C to perform water harvesting and radiative cooling involved electricity generation. In order to simulate the radiative cooling effect in the night sky, a water-cooling head with an inlet water temperature of 20 ºC was adopted and attached to the surface of the dual-functional coating layer plate [6]. The weight change of the SAWH-TEPG device was measured by a precision electronic balance (ME503TE, METLER TOLEDO) with a resolution of 1 mg which was in real-time communication with a computer. A voltmeter is used to measure the output voltage of the SAWH-TEPG device, and several temperature sensors are placed on the dual-functional coating layer, hot and cold sides of the TEPG module, and composite sorbent to measure the temperature changes during the water collecting and electricity generation process. As a contrast, another SAWH-TEPG device without MOF sorbent was also fabricated and tested to investigate the effect of the sorbent on the performance of the electricity generation.

For daytime proof-of-concept experiments, in prior to the experiments, the hybrid SAWH-TEPG device undergoes a thorough saturation stage in an environment of 90% RH and 20 ◦C overnight. The water harvesting experiments were firstly carried out in our laboratory under artificial lighting generated by a solar simulator with an irradiation intensity of 500, 750, and 1000 W m^{-2} (UHE-NS-100, SCIENCETECH, spot diameter of 100 mm × 1 cm), with uniformity of 10 %, temporal instability of 10 %, and spectral match classification of Class AAA. As a contrast, another SAWH-TEPG device without MOF sorbent was also fabricated and tested. The weight changes of the SAWH-TEPG device under different simulated sunlight intensity were measured by a precision electronic balance (ME503TE, METLER TOLEDO). The output voltage and the temperature evolution of the solar dual-functional coating layer, TEPG module, and sorbent were also recorded. Notably, during the experiments, the enclosure sidewalls of

the aluminum fins plate are opened to allow the free migration of the disported water vapor. All experiments of the SAWH-TEPG devices were carried out in an environment-controlled chamber and the swings of temperature and RH were kept below 0.5°C and 2%, measured by the thermo/hygrometer (HF335, Rotronic).

(ii) Outdoor experiments

The outdoor water harvesting experiments were implemented on the roof of our laboratory. The water and electricity co-generation experiment comprise two phases: night-time vapor adsorption and radiative cooling involved electricity generation; day-time solar-driving water harvesting, condensation and electricity co-generation. During night-time vapor adsorption, the experiment was first conducted on March 25, 2021, from 9:30 pm to 2:30 am (UTC+8) for radiative cooling enhanced water sorption and electricity generation at night. Prior to the first cycle, the MOF layer was heated and dehydrated through the desorption stage at 85 ºC for 5h. The dual-functional coating layer was positioned to face the clear sky to enable passive radiative cooling, thus reducing the MOF layer temperature. Transparent polyethylene film was used to suppress convective heat loss on the dual-functional coating layer of the device. Prior to the exposure to the clear sky, the container cover was wrapped in aluminum foil so that the MOF layer could equilibrate with the ambient air. Once the foil was removed, the MOF layer was exposed to the sky and performed vapor adsorption. The output voltage and the temperature evolution of the dual-functional coating layer, TEPG module, sorbent, environment temperature and RH were recorded through voltmeter, thermocouples and thermo/hygrometer (HF335, Rotronic). After the sorbent was saturated overnight, the device was sealed to prevent undesired water loss due to the RH swing, and the solar-driven water desorption and harvest was performed on March 26,

from 9:30 am to 2:30 pm (UTC+8). Ambient humidity and temperature conditions were recorded as described during the adsorption phase. In addition to the output voltage of the TEPG module and the temperatures of the TEPG module, sorbent and the dual-functional coating layer, the solar flux was measured by a pyranometer (TBQ-DL, Jinzhou Sunshine Technology), and the mass variation of the device during water capture and release processes were measured by using a precision electronic balance (ME503TE, METLER TOLEDO). Notably, the water collection performance of this device was also studied by measuring the mass gain for several nights between March and April.

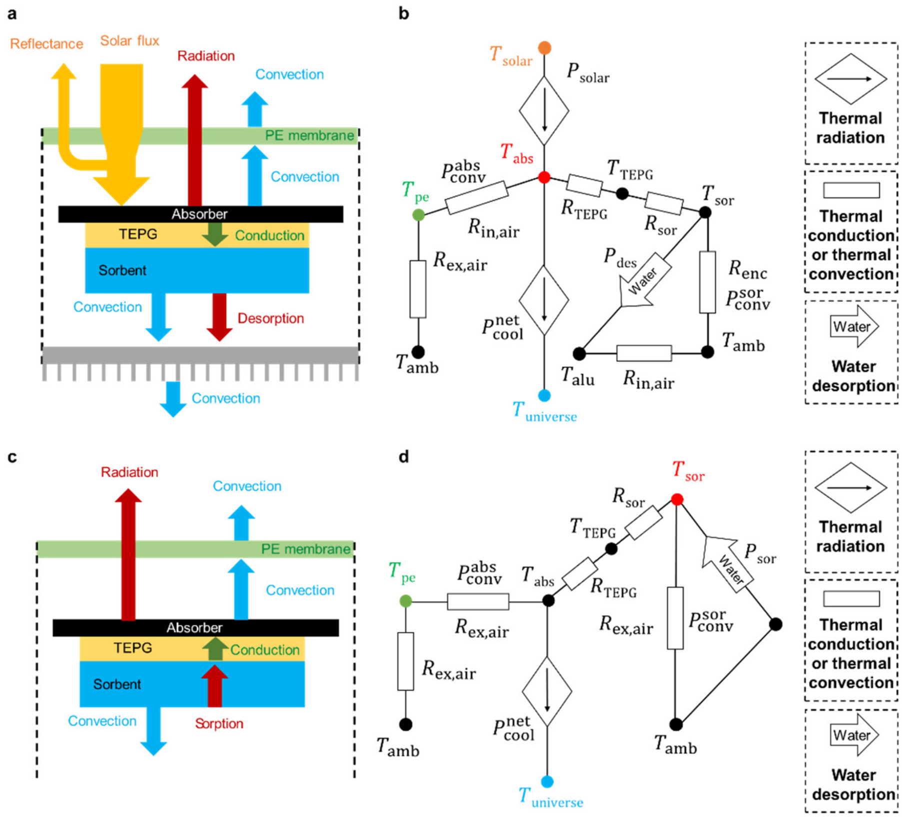

Supplementary Figure 1. (a) Energy balance model and (b) theoretical thermal equilibrium analysis of the hybrid SAWH-TEPG device during daytime. (c) Energy balance model and (d) theoretical thermal equilibrium analysis of the hybrid SAWH-TEPG device during nighttime.

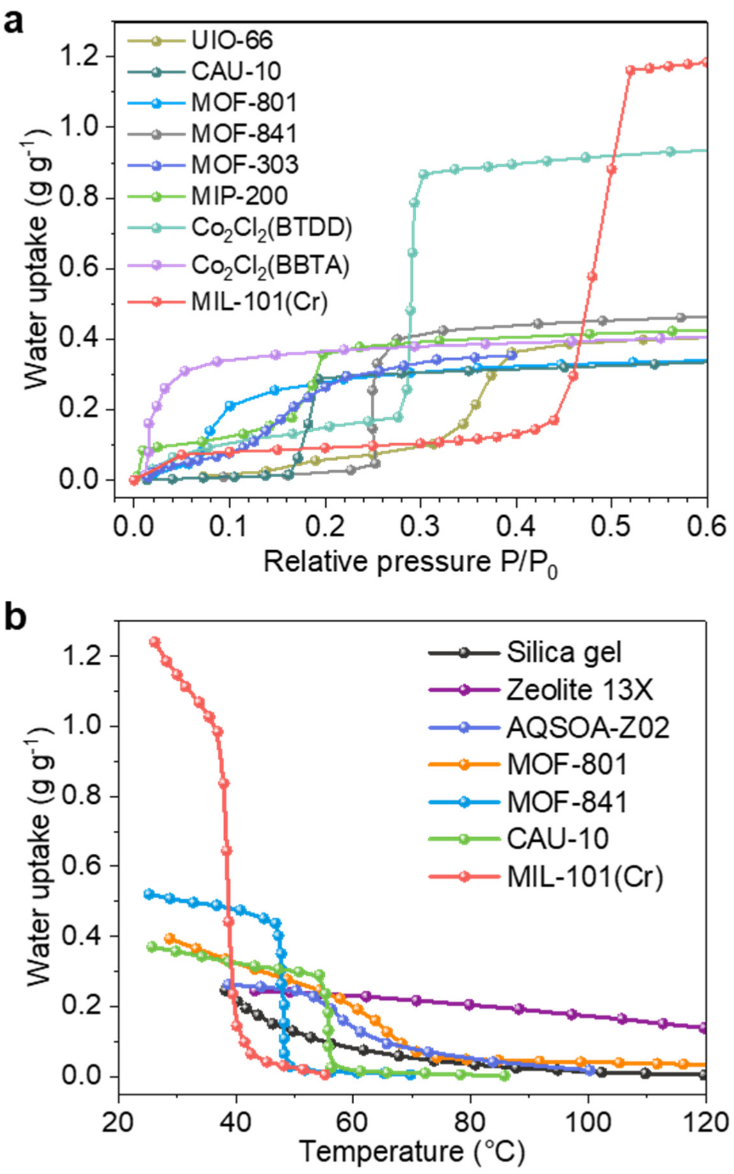

Supplementary Figure 2. (a) Water sorption of various sorbents at typical climates of 70% RH at 30 °C (~3.0 kPa) (b) The isothermal equilibrium curves of reported sorbents.[7-17]

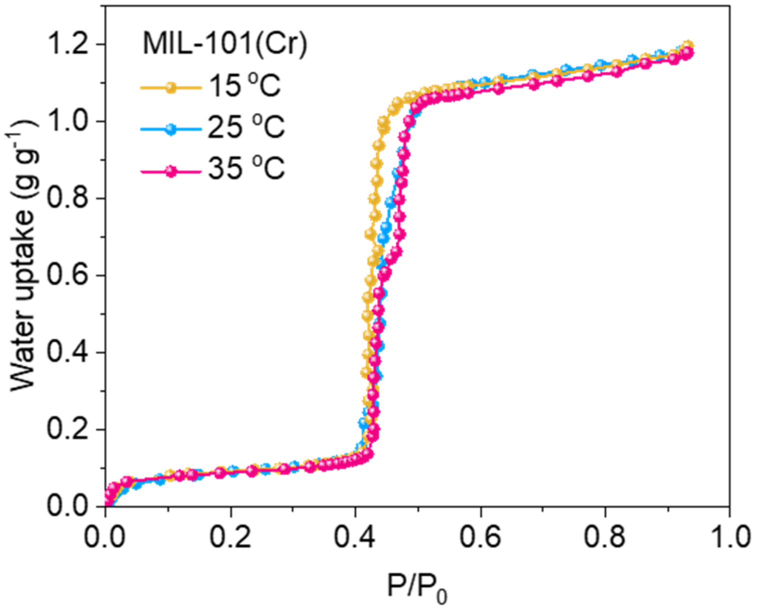

Supplementary Figure 3. Variable-temperature water vapor sorption isotherms of pristine MIL-101(Cr) powder at 15°C, 25°C, and 35°C, showing the temperature-insensitive characteristic.

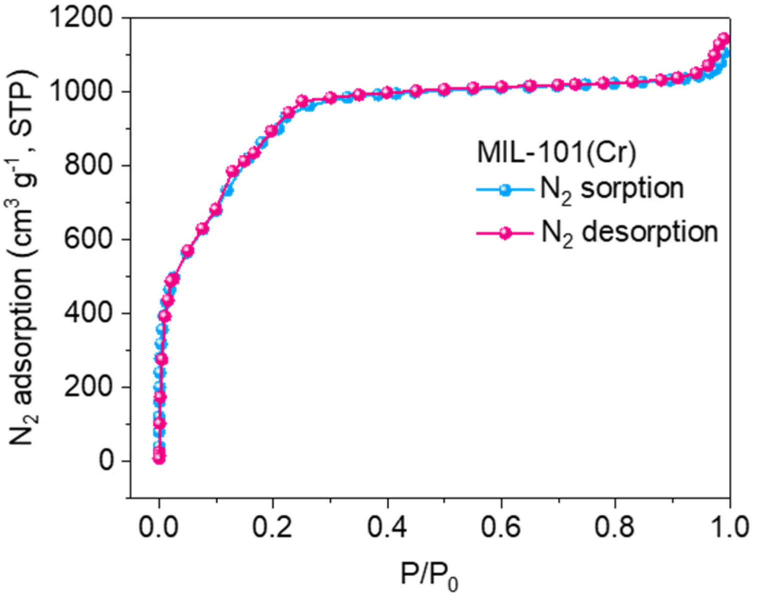

Supplementary Figure 4. N_2 adsorption isotherms of synthetic MIL-101(Cr) powder at 77K. The BET surface area is determined to be 3224.6 m^2 g^{-1}.

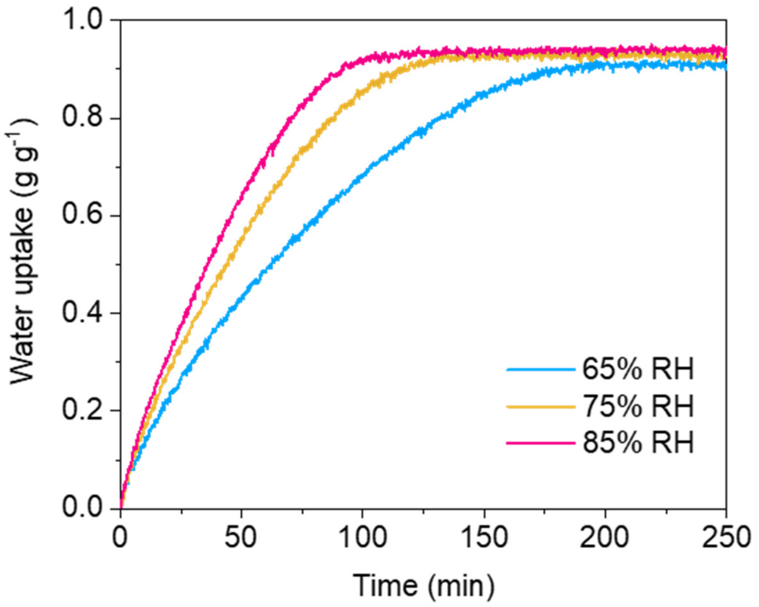

Supplementary Figure 5. Sorption kinetics of MIL-101(Cr)@CF composite at different RH.

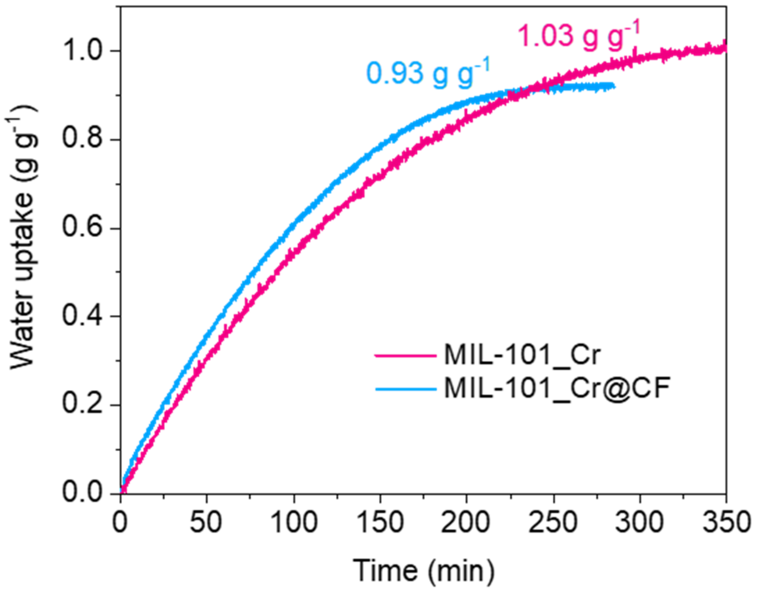

Supplementary Figure 6. Water uptake of MIL-101(Cr) powder and MIL-101(Cr)@CF composite samples at typical 25 ºC and 65% RH.

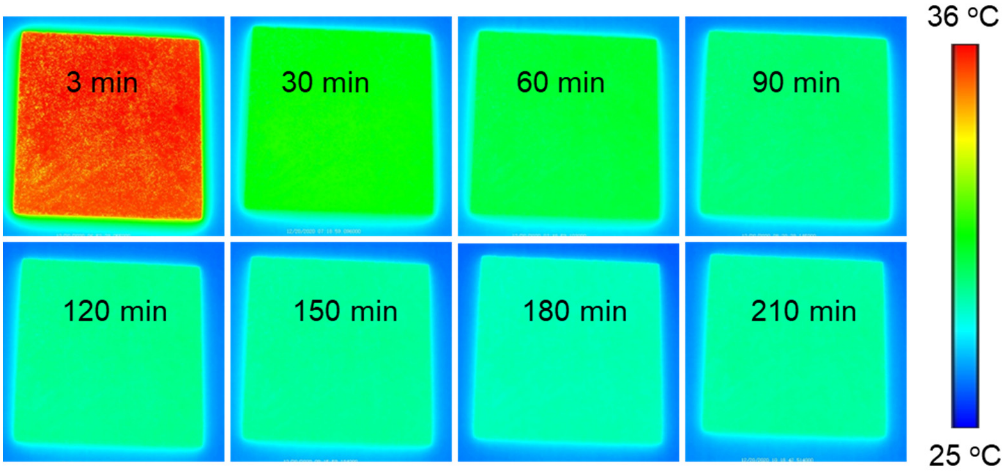

Supplementary Figure 7. IR images of MIL-101(Cr)@CF samples during moisture sorption process at different times.

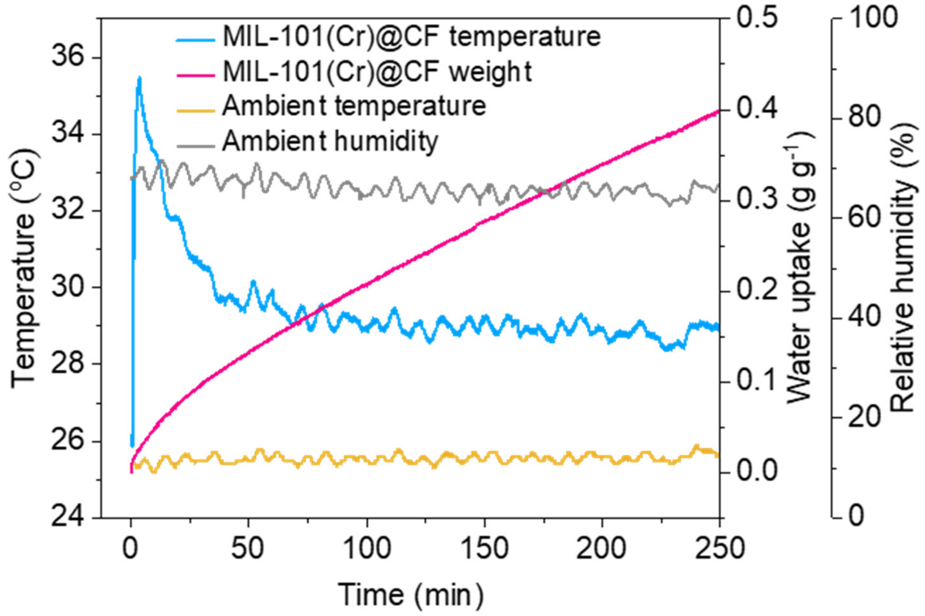

Supplementary Figure 8. Temperature evolution, water uptake of MIL-101(Cr)@CF samples and ambient condition during moisture sorption process.

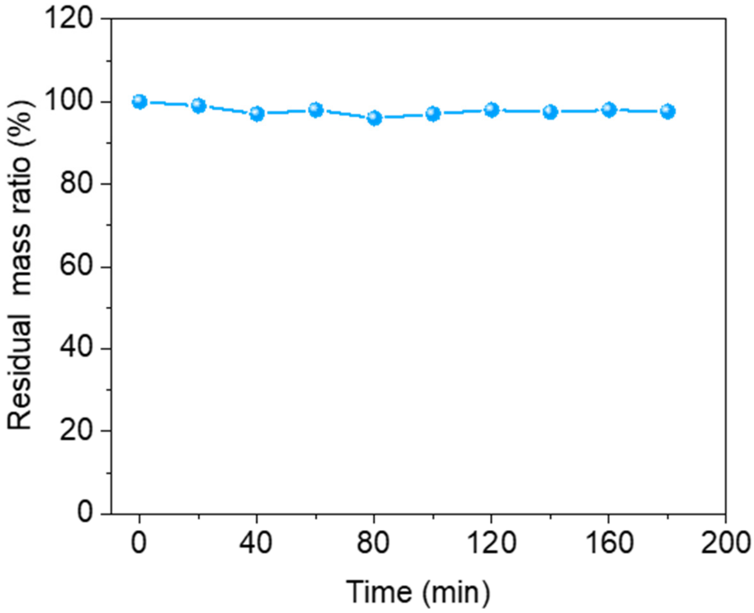

Supplementary Figure 9. Mass changes of MIL-101(Cr)@CF during shaky condition simulated by an 80 W ultrasonic cleaner.

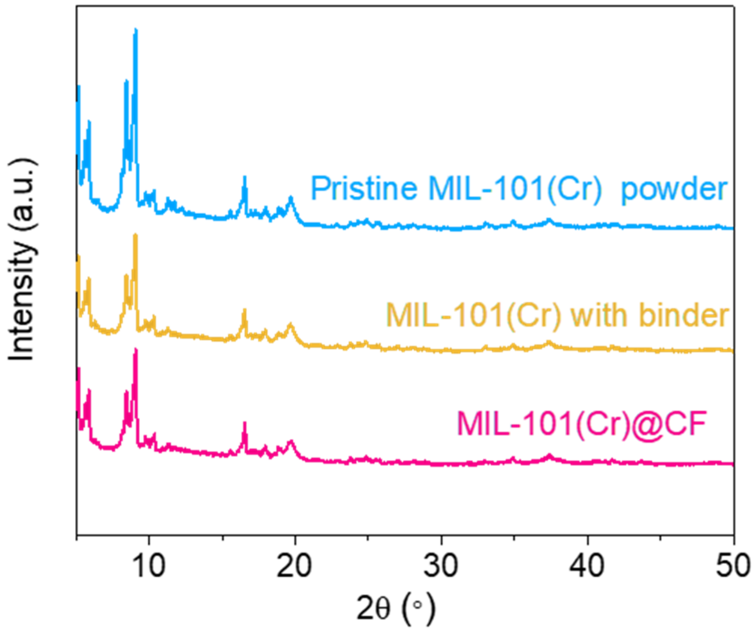

Supplementary Figure 10. Powder X-ray diffraction (PXRD) of pristine MIL-101(Cr) power, MIL-101(Cr) with binder, and MIL-101(Cr)@CF after 20 sorption-desorption cycles.

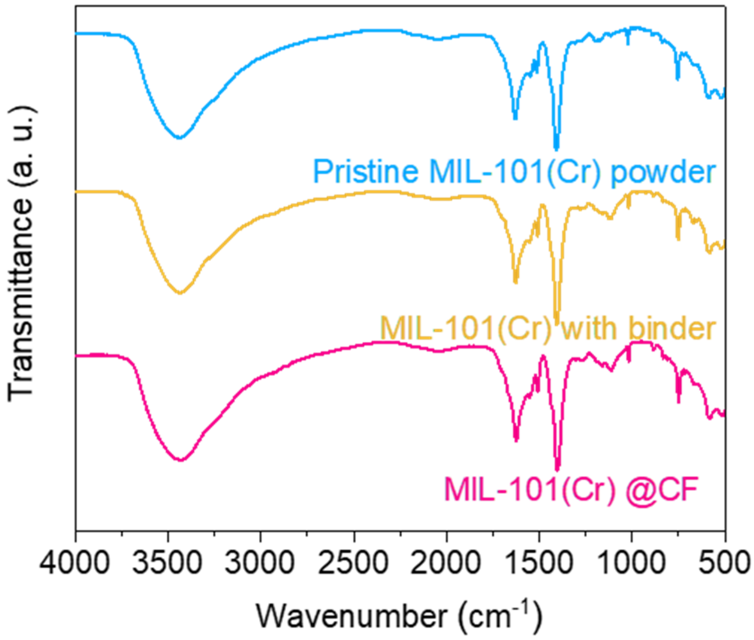

Supplementary Figure 11. FTIR patterns of pristine MIL-101(Cr) powder, MIL-101(Cr) with binder, and MIL-101(Cr)@CF after 20 sorption-desorption cycles.

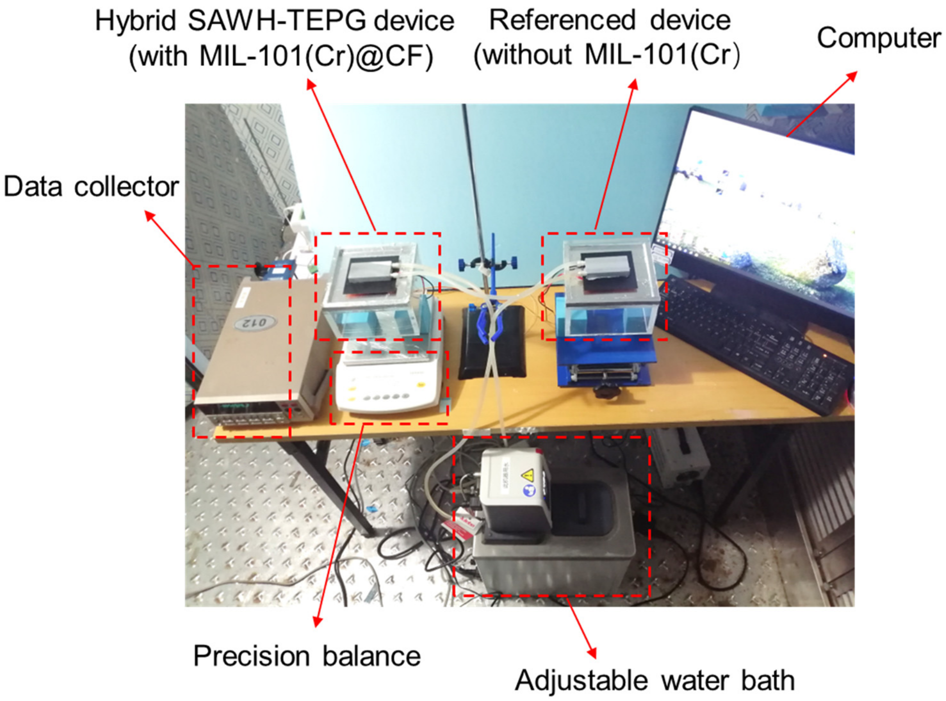

Supplementary Figure 12. Experimental setup of home-made test rig in the environment-controlled chamber.

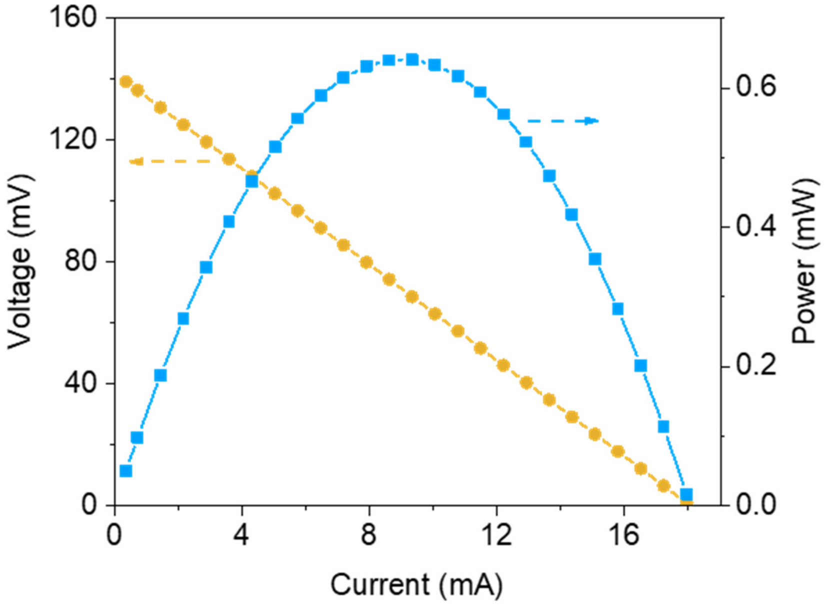

Supplementary Figure 13. Output voltage, current and power of the TEPG module, under the condition of 2 ºC temperature difference.

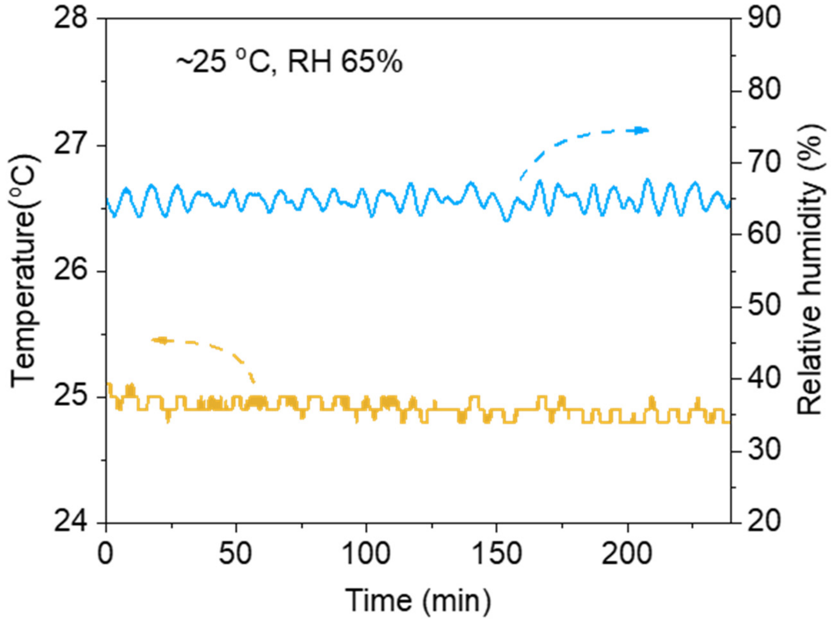

Supplementary Figure 14. Variation of ambient condition within a typical experimental period in the test chamber.

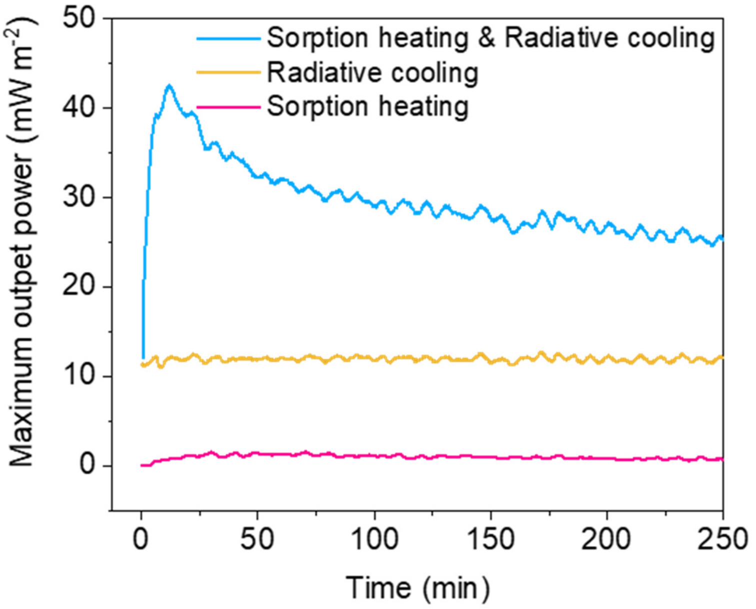

Supplementary Figure 15. Comparison of maximum output voltages of three different experimental devices.

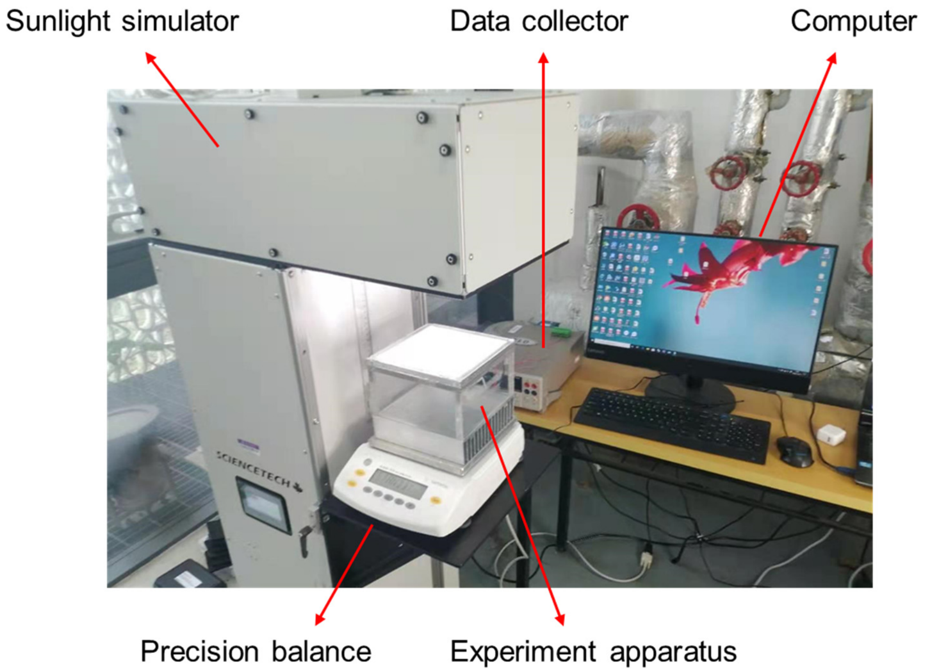

Supplementary Figure 16. Photograph of hybrid SAWH-TEPG device for indoor experiments under simulated sunlight.

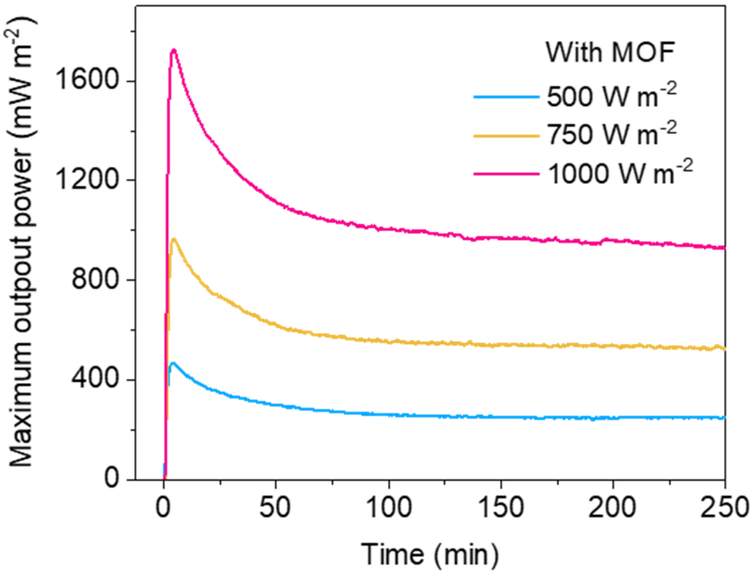

Supplementary Figure 17. Maximum output power densities of the hybrid SAWH-TEPG device under different simulated sunlight intensities.

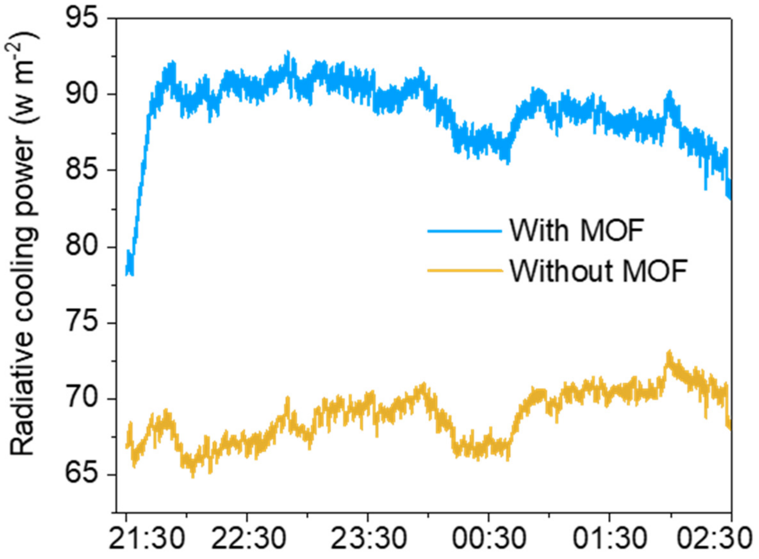

Supplementary Figure 18. Comparison of radiative cooling power of the hybrid SAWH-TEPG devices with MOF and the referenced device without MOF during nighttime.

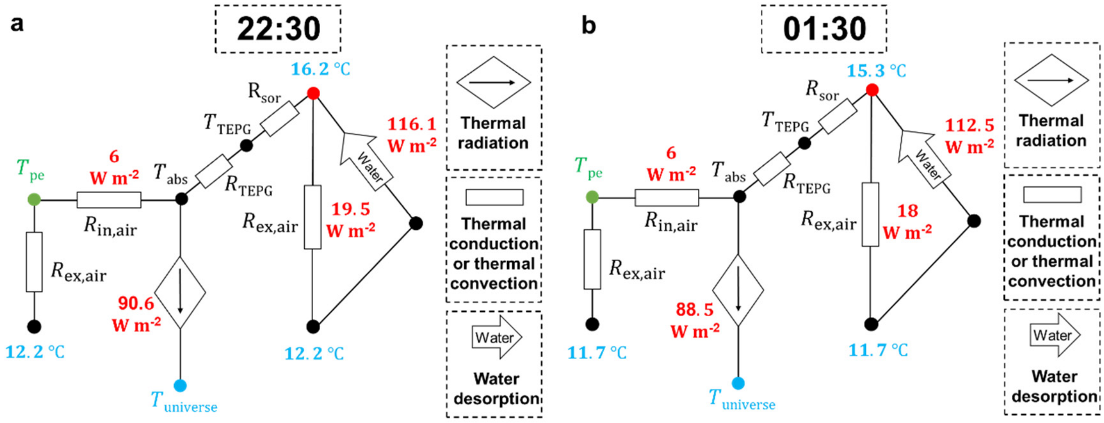

Supplementary Figure 19. Comparison of sorption heating power, radiative cooling power, and convective heat loss of the hybrid SAWH-TEPG devices at 22:30 pm and 1:30 am during nighttime.

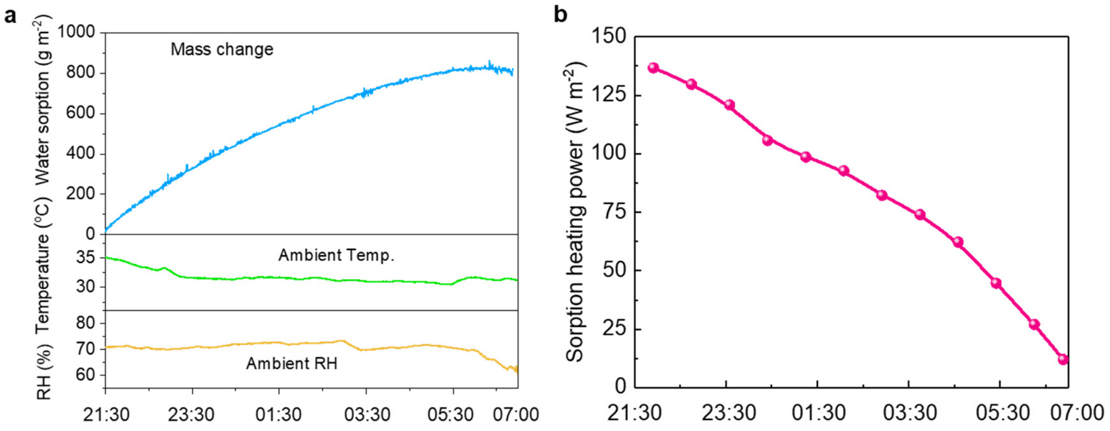

Supplementary Figure 20. (a) Water uptake, ambient temperature, and ambient RH changes of the device during the sorption process. (b) Sorption heating power of the sorbent

Note； Sorption heating power of the sorbent is calculated by the following equation:

$$P_{des} = \frac{\dot{m}_{\mathrm{MOF}} \cdot \Delta H_{\mathrm{H_2O}}}{M_{\mathrm{H_2O}}} \tag{20}$$

where $\dot{m}_{\mathrm{MOF}}$ is the mass change rate (water desorption rate) of the MOF sorbent, $\Delta H_{\mathrm{H_2O}}$ is the water desorption enthalpy (44 kJ $\mathrm{mol_{water}}^{-1}$), $M_{\mathrm{H_2O}}$ is the relative molecular mass of the water.

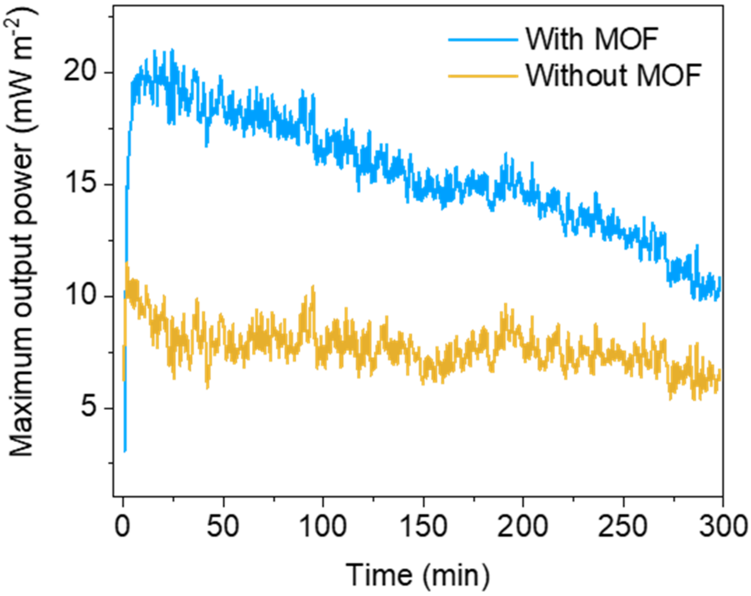

Supplementary Figure 21. Comparison of maximum output power of the hybrid SAWH-TEPG devices with MOF and the referenced device without MOF during nighttime.

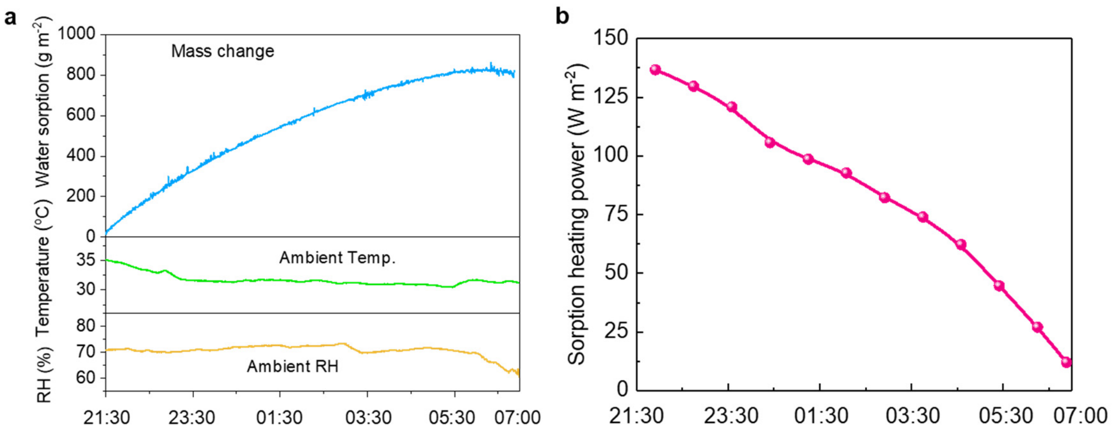

Supplementary Figure 22. (a) Theoretical thermal equilibrium results of the hybrid SAWH-TEPG device at (a) 12:30 pm and (b) 14:00 pm during daytime.

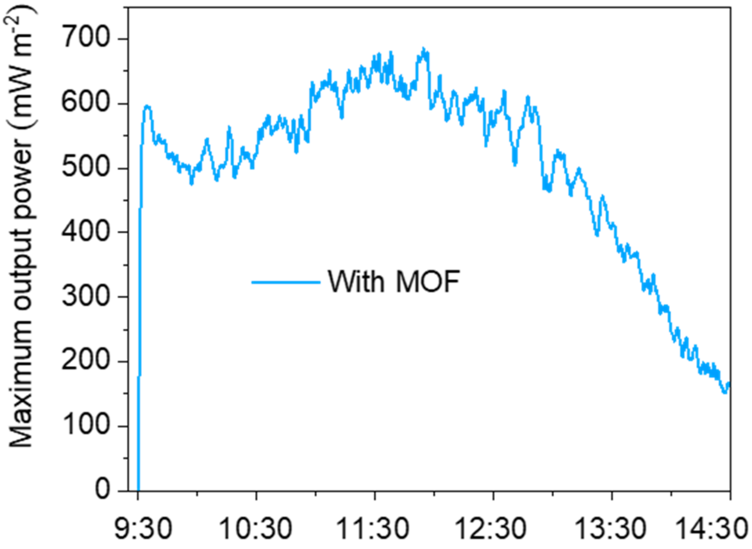

Supplementary Figure 23. Maximum output power of hybrid SAWH-TEPG device with MOF during daytime.

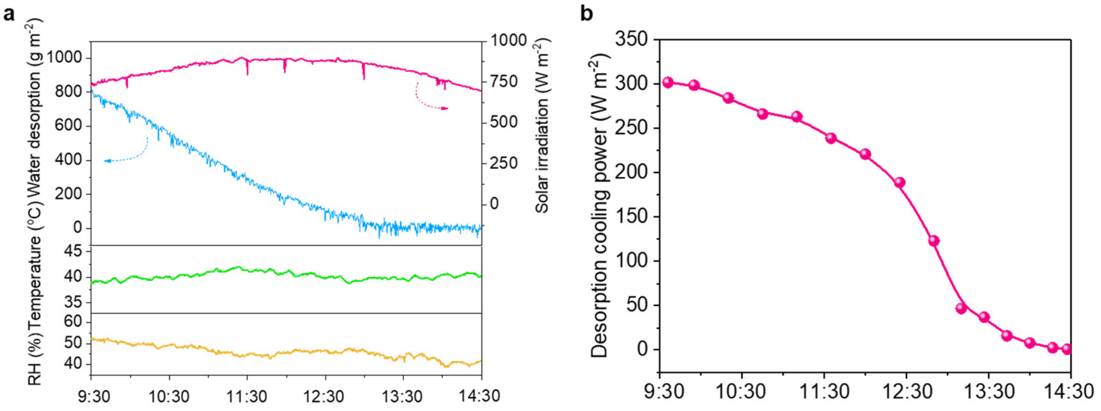

Supplementary Figure 24. (a) Mass, ambient temperature, and ambient RH changes of the device during the desorption process. (b) Desorption cooling power of the sorbent

Note: Desorption cooling power of the sorbent is calculated by the following equation:

$$P_{sor} = \frac{\dot{m}_{\mathrm{MOF}} \cdot \Delta H_{\mathrm{H_2O}}}{M_{\mathrm{H_2O}}} \quad (21)$$

where $\dot{m}_{\mathrm{MOF}}$ is the mass change rate (water sorption rate) of the MOF sorbent, $\Delta H_{\mathrm{H_2O}}$ is the water sorption enthalpy (44 kJ $\mathrm{mol_{water}}^{-1}$), $M_{\mathrm{H_2O}}$ is the relative molecular mass of water.

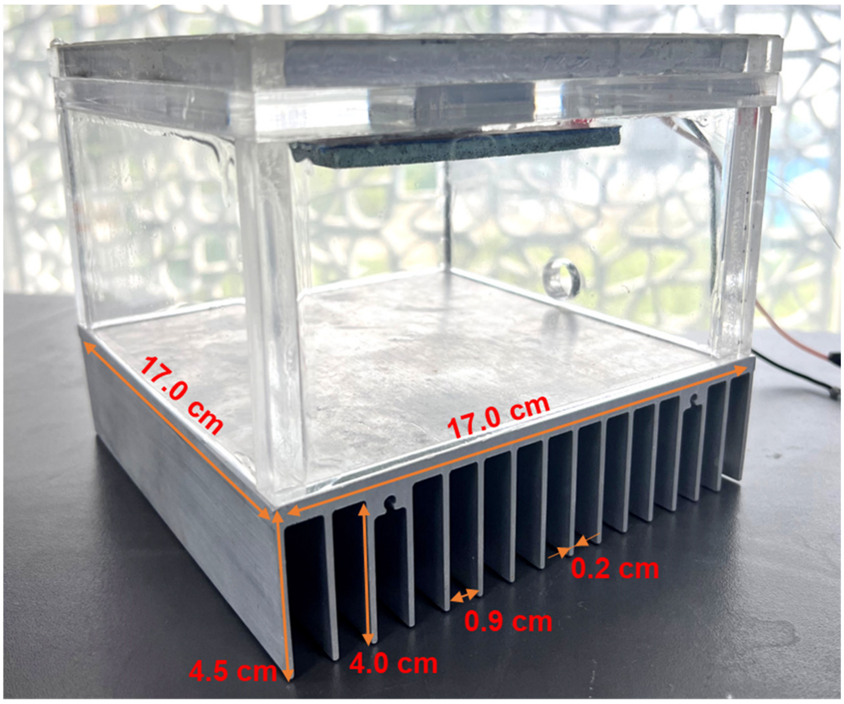

Supplementary Figure 25. Photograph of air-cooled condenser using aluminum flat fins

Note: The energy balance of air-cooled condenser during water condensation process can be expressed as following equation,

$$\frac{\mathrm{d}m_{\mathrm{H_2O}}}{dt}\Delta H_{\mathrm{H_2O}} = h\eta_0 A_{\mathrm{con}}(T_{\mathrm{con}} - T_{\mathrm{ambient}}) \quad (22)$$

where the m_{H2O} is the mass of water condensed on the air-cooled condenser, the ΔH_{H2O} is the condensation heat per gram water, the T_{con} represents the temperature of condenser, the h represents the natural heat convection coefficient estimated as 10 W m^{-2} K^{-1}), and the η_0 represents the overall fin surface efficiency, which is considered as 1 because natural heat convection is much slower than the thermal conduction of aluminum (272 W m^{-1} K^{-1}). According to the water harvesting rate of 150 g m^{-2} h^{-1}, the required surface area of condenser is 0.092 m^2 to keep a condensation temperature no more 1 ℃ higher than ambient. Therefore, we employed the aluminum radiator with fins as water condenser, whose heat exchange surface area is 0.247 m^2, provides enough heat exchange surface to efficiently dissipate the condensation heat release by water vapor.

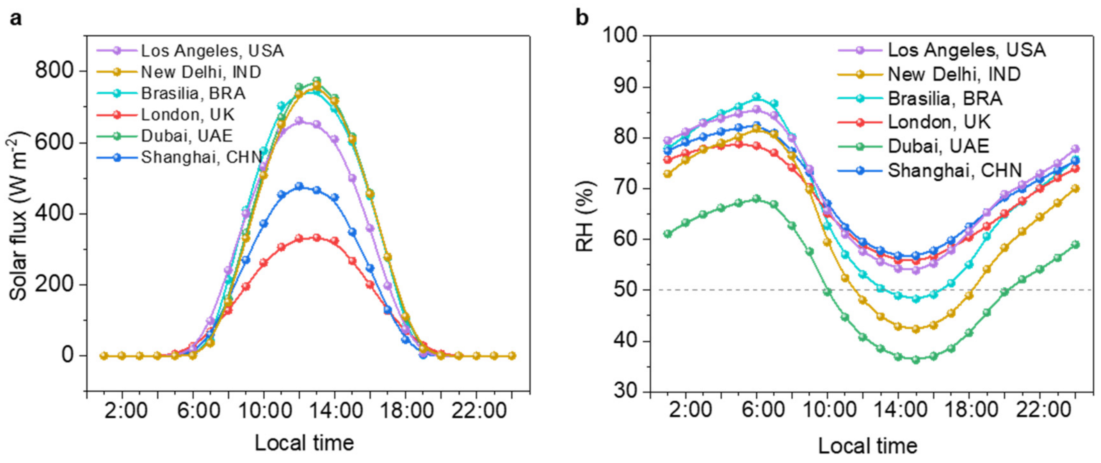

Supplementary Figure 26. (a) Variation of annual average solar irradiation intensity in one day. (b) Variation of annual average relative humidity in one day.

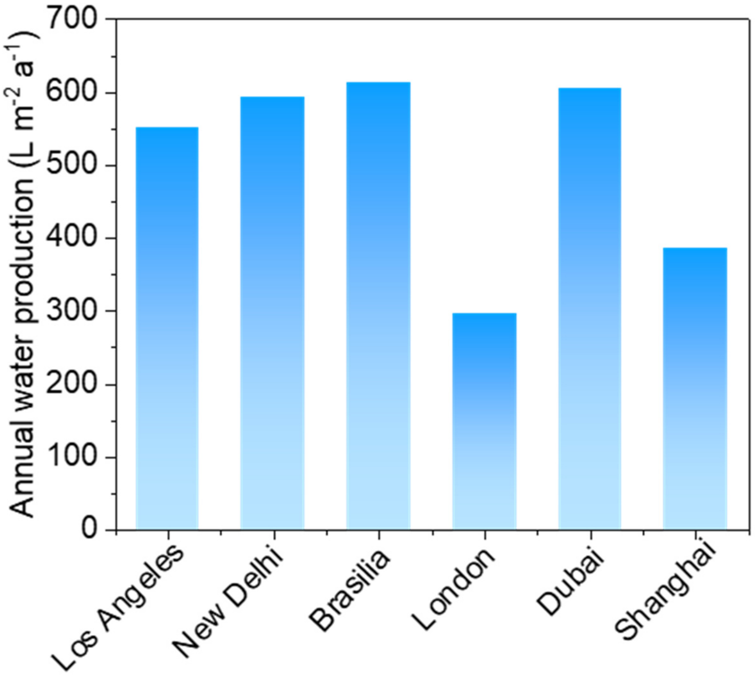

Supplementary Figure 27. Predicted annual water production of the SAWH-TEPG device in different cities.

Note: The predicted annual water production (M_{water}) of our device is calculated by the following equations:

$$M_{\text{water}} = 365 \cdot \eta \cdot Q_{\text{solar}} \tag{23}$$

$$Q_{\text{solar}} = \int_{t_0}^{t_{\text{end}}} \alpha \cdot A \cdot q_{\text{solar}} \mathrm{d}t \tag{24}$$

where η is the solar-driven water desorption efficiency, Q_{solar} is the input solar energy, α is the solar absorptance of solar collector (~0.95), A is the area of the solar absorber (0.1×0.1 m^2), and q_{solar} is the instantaneous solar irradiation intensity.

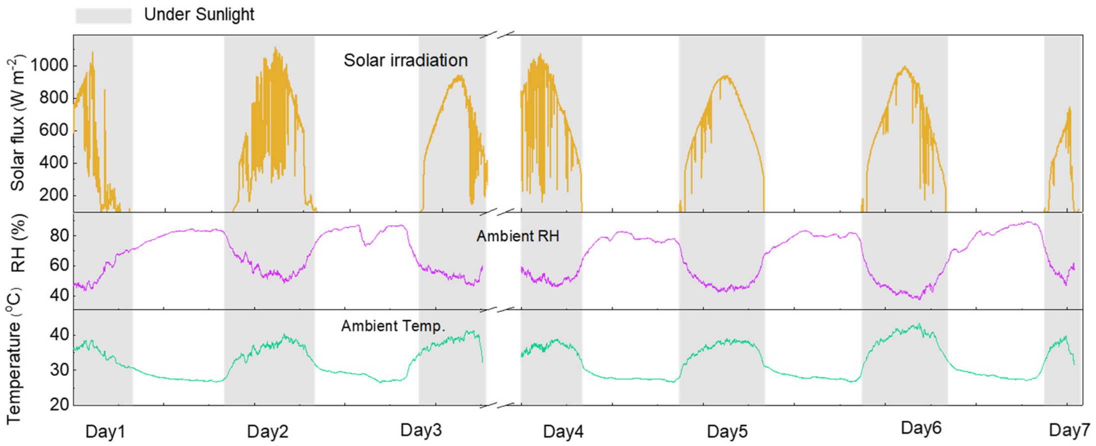

Supplementary Figure 28. Solar irradiation, air RH, and ambient temperature during a continuous week of outdoor experiments

Supplementary Table 1. Comparison of power density of different simultaneous water and electricity generation systems

		Daytime	Nighttime	Output type	
Solar intensity ($kW\ m^{-2}$)	Electricity generation Methods	Power density ($W\ m^{-2}$)	Power density ($mW\ m^{-2}$)		Ref.
1	Thermoelectric	0.4	-	Transient	[18]
1	Pyroelectric-piezoelectric	2.4×10^{-4}	-	Transient	[19]
1	Triboelectric	2.6×10^{-4}	-	Transient	[20]
1	Thermoelectric	0.175	-	Transient	[21]
1	Thermoelectric	0.4	-	Transient	[22]
1	Thermoelectric	0.22	-	Transient	[23]
0.9	Thermoelectric	0.22	-	Transient	[24]
1	Thermoelectric	0.072	-	Transient	[25]
1	Thermoelectric	0.5	-	Transient	[26]
-	Thermal resonant	0.203	< 1	Continuous	[27]
~0.9	Thermoelectric	0.52	6.6	Continuous	[28]
1	Thermoelectric	0.685	21	Continuous	This work

Supplementary References

1. Zhao, D., Aili, A., Zhai, Y., Xu, S., Tan, G., Yin, X,. Yang, R. Radiative sky cooling: Fundamental principles, materials, and applications. *Appl. Phys. Rev.* **6**, 021306 (2019).
2. Wang, T., Wu, Y., Shi, L., Hu, X., Chen, M., Wu, L. A structural polymer for highly efficient all-day passive radiative cooling. *Nat. commun.* **12**, 365 (2021).
3. Raman, A.P., Li, W., Fan, S. Generating Light from Darkness - ScienceDirect. *Joule* **3**, 11(2019).
4. Zhao, M., Yuan, K., Wang, Y., Li, G., Guo, J., Gu, L., Hu, W., Zhao, H., and Tang, Z. Metal–organic frameworks as selectivity regulators for hydrogenation reactions. *Nature* **539**, 76-80 (2016).
5. Wang, C., Hua, L., Yan, H., Li, B., Tu, Y., and Wang, R. A thermal management strategy for electronic devices based on moisture sorption-desorption processes. *Joule* **4**, 435-447(2020).
6. Raman, A.P., Li, W., and Fan, S. Generating light from darkness. *Joule* **3**, 2679-2686(2019).
7. Aristov, Y.I., Tokarev, M.M., Freni, A., Glaznev, I.S., and Restuccia, G. Kinetics of water adsorption on silica Fuji Davison RD. *Microporous Mesoporous Mater.* **96**, 65-71(2006).
8. Cortés, F., Chejne, F., Carrasco-Marín, F., Moreno-Castilla, C., and Pérez-Cadenas, A. Water adsorption on zeolite 13X: comparison of the two methods based on mass spectrometry and thermogravimetry. *Adsorption* **16**, 141-146 (2010).
9. De Lange, M.F., Verouden, K.J., Vlugt, T.J., Gascon, J., and Kapteijn, F. Adsorption-driven heat pumps: the potential of metal–organic frameworks. *Chem. Rev.* **115**, 12205-12250 (2015).
10. Fathieh, F., Kalmutzki, M.J., Kapustin, E.A., Waller, P.J., Yang, J., and Yaghi, O.M. Practical water production from desert air. *Sci. adv.* **4**, eaat3198 (2018).
11. Fröhlich, D., Henninger, S.K., and Janiak, C. Multicycle water vapour stability of microporous breathing MOF aluminium isophthalate CAU-10-H. *Dalton Trans.* **43**, 15300-15304(2014).
12. Furukawa, H., Gandara, F., Zhang, Y.-B., Jiang, J., Queen, W.L., Hudson, M.R., and Yaghi, O.M. Water adsorption in porous metal–organic frameworks and related materials. *J. Am. Chem. Soc.* **136**, 4369-4381(2014).

13. Ghosh, P., Colón, Y.J., and Snurr, R.Q. Water adsorption in UiO-66: the importance of defects. *Chem. Commun.* **50**, 11329-11331(2014).
14. Kim, H., Yang, S., Rao, S.R., Narayanan, S., Kapustin, E.A., Furukawa, H., Umans, A.S., Yaghi, O.M., and Wang, E.N. Water harvesting from air with metal-organic frameworks powered by natural sunlight. *Science* **356**, 430-434(2017).
15. Rieth, A.J., Wright, A.M., Rao, S., Kim, H., LaPotin, A.D., Wang, E.N., and Dincă, M. Tunable metal–organic frameworks enable high-efficiency cascaded adsorption heat pumps. *J. Am. Chem. Soc.* **140**, 17591-17596(2018).
16. Rieth, A.J., Yang, S., Wang, E.N., and Dinca, M. Record atmospheric fresh water capture and heat transfer with a material operating at the water uptake reversibility limit. *ACS Cent. Sci.* **3**, 668-672(2017).
17. Wang, S., Lee, J.S., Wahiduzzaman, M., Park, J., Muschi, M., Martineau-Corcos, C., Tissot, A., Cho, K.H., Marrot, J., and Shepard, W. A robust large-pore zirconium carboxylate metal–organic framework for energy-efficient water-sorption-driven refrigeration. *Nat. Energy* **3**, 985-993(2018).
18. Zhang, Y., Ravi, S. K., and Tan, S. C. Food-derived carbonaceous materials for solar desalination and thermo-electric power generation. *Nano Energy* **65**, 104006(2019).
19. Zhu, L., Gao, M., Peh, C. K. N., Wang, X., and Ho, G. W. Self-contained monolithic carbon sponges for solar-driven interfacial water evaporation distillation and electricity generation. *Adv. Energy Mater.* **8**, 1702149(2018).
20. Gao, M., Peh, C. K., Phan, H. T., Zhu, L., and Ho, G. W. Solar absorber gel: localized macro-nano heat channeling for efficient plasmonic Au nanoflowers photothermic vaporization and triboelectric generation. *Adv. Energy Mater.* **8**, 1800711(2018).
21. Jiang, H., Ai, L., Chen, M., and Jiang, J. Broadband nickel sulfide/nickel foam-based solar evaporator for highly efficient water purification and electricity generation. *ACS Sustain. Chem. Eng.* **8**, 10833-10841(2020).
22. Zhu, L., Ding, T., Gao, M., Peh, C. K. N., and Ho, G. W. Shape conformal and thermal insulative organic solar absorber sponge for photothermal water evaporation and thermoelectric power generation. *Adv. Energy Mater.* **9**, 1900250(2019).
23. Zhang, X., Gao, W., Su, X., et al. Conversion of solar power to chemical energy

based on carbon nanoparticle modified photo-thermoelectric generator and electrochemical water splitting system. *Nano Energy* **48**, 481-488(2018).

24. Zhang, Q., Chen, S., Fu, Z., Yu, H., and Quan, X. Temperature-difference-induced electricity during solar desalination with bilayer MXene-based monoliths. *Nano Energy* **76**, 105060(2020).
25. Li, N., Yang, D. J., Shao, Y., et al. Nanostructured black aluminum prepared by laser direct writing as a high-performance plasmonic absorber for photothermal/electric conversion. *ACS Appl. Mater. Inter.* **13**, 4305-4315(2021).
26. Liu, X., Mishra, D. D., Li, Y., Gao, L., Peng, H., Zhang, L., and Hu, C. Biomass-derived carbonaceous materials with multichannel waterways for solar-driven clean water and thermoelectric power generation. *ACS Sustain. Chem. Eng.* **9**, 4571-4582(2021).
27. Cottrill, A. L., Liu, A. T., Kunai, Y., et al. Ultra-high thermal effusivity materials for resonant ambient thermal energy harvesting. *Nat. Commun.* **9**, 664(2018).
28. Yang, K., Pan, T., Pinnau, I., Shi, Z., and Han, Y. Simultaneous generation of atmospheric water and electricity using a hygroscopic aerogel with fast sorption kinetics. *Nano Energy* **78**, 105326(2020).
